# Reconsidering the evidence for learning in single cells

**DOI:** 10.7554/eLife.61907

**Published:** 2021-01-04

**Authors:** Samuel J Gershman, Petra EM Balbi, C Randy Gallistel, Jeremy Gunawardena

**Affiliations:** 1 Department of Psychology and Center for Brain Science, Harvard UniversityCambridgeUnited States; 2 Center for Brains, Mind and Machines, MITCambridgeUnited States; 3 Department of Systems Biology, Harvard Medical SchoolBostonUnited States; 4 Rutgers Center for Cognitive Science, Rutgers University at New BrunswickNew BrunswickUnited States; University of Texas at AustinUnited States; University of Texas at AustinUnited States

**Keywords:** single cell, learning, Paramecium, Pavlovian conditioning

## Abstract

The question of whether single cells can learn led to much debate in the early 20th century. The view prevailed that they were capable of non-associative learning but not of associative learning, such as Pavlovian conditioning. Experiments indicating the contrary were considered either non-reproducible or subject to more acceptable interpretations. Recent developments suggest that the time is right to reconsider this consensus. We exhume the experiments of Beatrice Gelber on Pavlovian conditioning in the ciliate *Paramecium aurelia*, and suggest that criticisms of her findings can now be reinterpreted. Gelber was a remarkable scientist whose absence from the historical record testifies to the prevailing orthodoxy that single cells cannot learn. Her work, and more recent studies, suggest that such learning may be evolutionarily more widespread and fundamental to life than previously thought and we discuss the implications for different aspects of biology.

## Introduction

The emergence of learning was a major event in evolutionary history, allowing organisms to adapt to their environment on time scales much faster than genetic selection. But when exactly did learning emerge, and in what form? The vast majority of research on learning has been conducted in multicellular organisms, mostly vertebrates with complex nervous systems. This focus has led to biological models of learning based on synaptic plasticity (Martin et al., 2000). A plastic synapse, also known as a Hebbian synapse, changes its conductance in response to the close temporal pairing of pre- and post-synaptic spiking. In spike-timing-dependent plasticity, this temporal pairing is measured in milliseconds. A change in synaptic conductance is a change in the magnitude and sign (excitatory/inhibitory) of the postsynaptic currents produced by a presynaptic spike. In connectionist models of learning and memory, synaptic conductances are represented by signed connection weights with a scalar effect on the postsynaptic signal.

However, unicellular organisms capable of complex behaviors (see Dexter et al., 2019; Jennings, 1906) existed for over a billion years before the appearance of multicellular organisms (Wright and Lynn, 1997). This raises the question of whether unicellular organisms are also capable of learning, despite lacking nervous systems, as an evolutionary solution to the information processing challenges common to all living systems. The answer to this question has the potential to profoundly reshape our understanding of learning in multicellular organisms, as we discuss below.

To avoid ambiguity, we begin by fixing some terminology. Broadly construed, learning refers to any persistent and adaptive modification of an organism’s behavior as a function of its experience (see Barron et al., 2015, for further discussion). This behaviorally focused definition avoids reference to underlying mechanism at the cost of confounding a variety of processes (e.g. maturation, fluctuating motivational states) that we would not wish to include (Domjan, 2014). For that reason, it is common to include an appeal to an hypothesized mechanism. In empiricist philosophy, behaviorist psychology and connectionist cognitive science, the hypothesized mechanism is the formation of associations (philosophy and psychology), connections (connectionism) and alterations in the strengths of plastic synapses (neuroscience). The information-processing tradition in cognitive science, sometimes called the computational theory of mind, by contrast, conceives of learning as the extraction of information from sensory experience and the storage of that information in a memory (Gallistel and King, 2011). The memory is the channel by which information extracted from present experience is communicated to the computational operations that inform behavior in the indefinite future. The difference in the two theoretical frameworks can be summarized as saying that the first approach focuses on the acquisition of information and the second approach focuses on the information itself.

In this article, we will focus on a canonical form of learning, *Pavlovian conditioning* (Pavlov, 1927), while acknowledging the evidence for other forms of learning in unicellular organisms (e.g. habituation; Wood, 1969; Tang and Marshall, 2018). In Pavlovian conditioning, an initially neutral stimulus (the conditioned stimulus, or CS) comes to elicit a conditioned response (CR) when it reliably predicts the occurrence of a motivationally attractive or aversive stimulus (the unconditioned stimulus, or US). Despite its apparent simplicity, Pavlovian conditioning is a sophisticated form of learning that eludes a simple characterization in terms of association formation between stimuli (Rescorla, 1988; Gallistel and Gibbon, 2002; Gershman et al., 2010), which is the standard textbook story. It is an open question whether unicellular organisms exhibit a comparable level of sophistication in their learning capabilities. We note that the question of learning, and especially of Pavlovian conditioning, in non-neural organisms continues to elicit both interest and controversy (Eisenstein, 1975; Reid et al., 2016; Markel, 2020a; Gagliano et al., 2020; Gagliano et al., 2016; Markel, 2020b; Baluška and Levin, 2016).

Pavlovian conditioning is particularly interesting for our purposes because of the prevailing theory that it is mediated by synaptic plasticity. This theory has been criticized on several grounds: that synaptic plasticity cannot account for the behavioral features of Pavlovian conditioning (Gallistel and Matzel, 2013); that it is too unstable to implement long-term memory storage due to molecular turnover (Crick, 1984; Mongillo et al., 2017); that it can be experimentally dissociated from behavioral measures of memory (Chen et al., 2014; Ryan et al., 2015); and that behaviorally relevant information is not stored in a readable format (Gallistel, 2017). Furthermore, a synaptic memory substrate requires that computations operate via the propagation of spiking activity, incurring an energetic cost roughly 13 orders of magnitude greater than the cost incurred if the computations are implemented using intracellular molecules (Gallistel, 2017).

As an alternative (or complement), it has been proposed that memory may be stored using a cell-intrinsic substrate, such as polynucleotide sequences (e.g. RNA), post-translational histone modifications, or DNA methylation patterns (Landauer, 1964; Crick, 1984; Day and Sweatt, 2010; Abraham et al., 2019; Gräff and Tsai, 2013; Gallistel, 2017). These theories posit a mapping from experienced quantities, like the duration of the interval between the onset of the conditioned stimulus and the onset of the unconditioned stimulus, to the hypothesized changes in molecular level structures that encode them. The biochemical processes that produce these changes in response to relevant synaptic input and that later convert the encoded information to appropriately timed output signals remain unknown, although recent work revealing a CamKII-dependent tunable event timer provides a possible biochemical mechanism for interval timing (Thornquist et al., 2020).

The possibility of a cellular-level mechansim for storing acquired information with delayed behaivoral consequences is exciting from an evolutionary perspective because it suggests that the mechanisms for memory storage in complex multicellular organisms may have been inherited from much simpler organisms, possibly even protozoa, that share the same intracellular molecular repertoire. Synaptic plasticity is clearly not an option for protozoa, so if evolution hit upon a way to implement learning in these organisms, it is natural to conjecture that such a mechanism would be conserved across phyla, given its computational and energetic advantages. A convincing demonstration of Pavlovian conditioning in a unicellular organism would render less surprising recent results that imply that the encoding of the CS-US interval in Pavlovian eyeblink conditioning is intrinsic to the cerebellar Purkinje cell (Johansson et al., 2014).

At present, this evolutionary argument is speculative, and comparative studies have often focused on animals (Ginsburg and Jablonka, 2010). Moreover, the question is complicated by the fact that cell-intrinsic mechanisms interact in complex ways with synaptic mechanisms in multicellular organisms. The most well-established theory is that cell-intrinsic mechanisms, such as histone modification and DNA methylation, serve an enabling rather than a storage function (Kandel, 2001; Lisman et al., 2018). By regulating gene expression, they enable the synthesis of synaptic plasticity-related proteins. This type of theory is clearly not applicable to memory storage in single cells, which lack synapses.

Before we can begin addressing the molecular storage mechanisms in single cells, we must address a prerequisite question: do single cells learn? And if so, what exactly are they capable of learning? This question has been tangled in controversy since the early 20th century. In this paper, we chart the history of past attempts to address the single cell learning question, focusing in particular on the remarkable, and largely forgotten, studies of Beatrice Gelber. At the time of their publication, these studies were criticized for failing to rule out various confounding explanations. We revisit these criticisms, arguing that some of them are misplaced, while some of them can be reinterpreted in a more positive light. In our concluding remarks, we discuss some broader implications for the cell biology of learning.

## Historical background

At the turn of the 20th century, the study of protozoa was given a jolt with the publication of the book *Behavior of the Lower Organisms* by the zoologist Herbert Spencer Jennings. At that time, an influential view, championed by the physiologist Jacques Loeb (see Pauly, 1981), held that protozoan behavior was driven by various ‘tropisms’ (heliotropism, galvanotropism, chemotropism, geotropism, etc.). The concept of a tropism was inherited from the study of movement in plants, where it was observed that some plants reoriented leaf surfaces such that the incident light was symmetric (a form of heliotropism). Loeb and his students applied this concept more broadly to algae and animals, arguing that many movements could be conceptualized as reactions to an asymmetrically impinging force (e.g. light, electrical currents, chemical gradients, gravity), resulting in either approach or avoidance. Like Loeb, Jennings believed that there were deep connections between patterns of animal behavior and those of simpler organisms. However, they understood the nature of these connections, and the nature of behavior itself, quite differently. Unlike Loeb (who was essentially a behaviorist in his sympathies), Jennings sought mechanistic ‘sensorimotor’ explanations of behavior, on the belief that the mechanisms in animals were an elaborated form of similar mechanisms found in simpler organisms.

Most importantly for our purposes, Jennings, 1906 argued that one of the relevant mechanisms underlying protozoan behavior was learning. Because tropisms were typically conceptualized as unlearned stimulus-response mappings, the learning hypothesis was problematic for Loeb’s tropism-based theory of protozoan behavior. For example, Jennings observed that repeated aversive stimulation of the ciliate *Stentor roeseli* resulted in a characteristic sequence of distinct avoidance behaviors (resting, bending away, ciliary alteration, contraction, and detachment). This sequence could be interpreted as an elementary form of learning, in that the same stimulus came to elicit a different response. Jennings recognized that this implied a change internal to the organism. The change, he argued, was adaptive, a key feature of learning in ‘higher’ organisms:

"The essential point seems to be that after experience the organism reacts in a more effective way than before. The change is regulatory, not merely haphazard." (Jennings, 1906, p. 178).

On this score, Jennings took pains to rule out the hypothesis that the sequence reflected some form of fatigue.

Jennings’ study of elementary learning was largely discredited among the experts because of claims of non-reproducibility (Reynierse and Walsh, 1967). However, this was likely the result of experimental failings, most glaringly the fact that Reynierse and Walsh used a different species of *Stentor*, and recent experimental work with the correct species has vindicated Jennings (Dexter et al., 2019). The reception of Jennings’ work on *S. roeseli* illustrates the prevailing tendency to discount complex behaviour in unicellular organisms, to the point of being less critical of experiments which give the expected result. It is also a reminder that even if learning is phylogenetically widespread it still needs to be studied in the ecological context of a particular organism: what one species of *Stentor* learns may be irrelevant to another.

Jennings arrived at some of the same ideas about associative learning that were concurrently being investigated by Ivan Pavlov in dogs, in particular the idea that the response to an aversive or appetitive stimulus could be ‘transferred’ to a neutral stimulus by virtue of an association between the two stimuli. Nonetheless, Jennings had little evidence for this claim. It was left to later scientists to fill the gap.

Day and Bentley, 1911 placed *Paramecia* (another ciliate) into a capillary tube, observing that the *Paramecia* turned and eventually reversed direction. With repeated trials, the *Paramecia* increased the speed with which they achieved reversal (see also Smith, 1908). Inspired by Day and Bentley, French, 1940 used a larger capillary tube, allowing easier turning, and found that over the course of trials the *Paramecia* became faster at escaping from the tube by swimming downwards. This pattern has been interpreted in terms of associative learning (e.g. Huber et al., 1974), based on the hypothesis that contact with the tube walls is aversive, so that the *Paramecium* forms an association between its swimming actions and relief from the aversive stimulus. However, Hinkle and Wood, 1994, using *Stentor* as their subjects, presented evidence that the learning effect may be confounded with the orientation of the tube: the subjects increased the frequency of downward swimming even when the escape was from the upper end of the tube. More recently, Armus et al., 2006 showed that *Paramecia* learned to associate the location of cathodal stimulation (which exerted an attractive effect) with a light-conditioned stimulus. Other studies used aversive unconditioned stimuli such as shock or heat (Bramstedt, 1935; Soest, 1937; Hennessey et al., 1979), but the results were inconsistent (Best, 1954; Mirsky and Katz, 1958) and beset by alternative interpretations, such as changes in the chemical composition of the medium (Grabowski, 1939).

Taken together, these studies leave an equivocal picture of single-cell learning capabilities. Moreover, even those studies that successfully demonstrated learning often did not systematically investigate the parameters governing the strength of the effect, its retention, or any hypothesis about the underlying mechanism.

**Figure 1.**
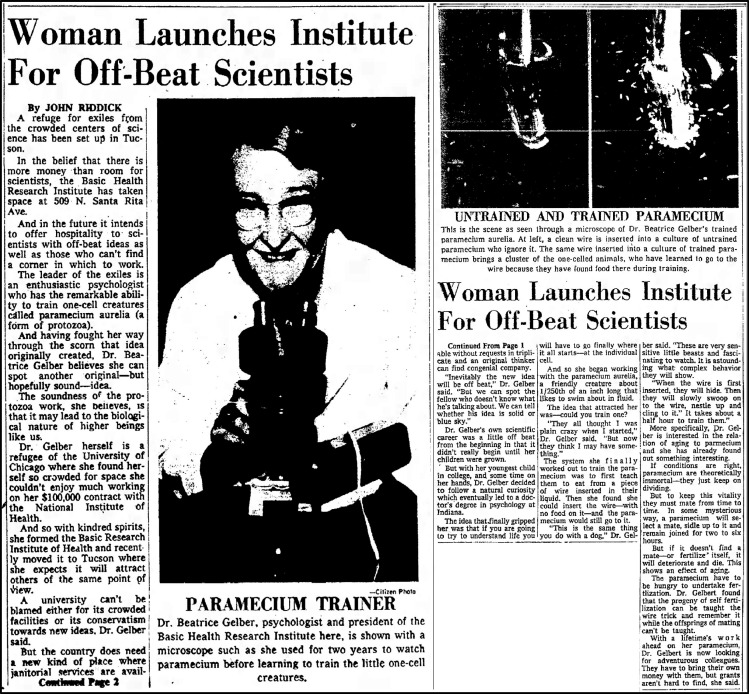
News story about Beatrice Gelber from the Tucson Daily Citizen October 19, 1960.

We next turn to one of the most systematic programs of research on learning in *Paramecia*—one which ignited considerable controversy during its heyday, but was eventually consigned to the scientific waste-bin. The remarkable scientist who single-handedly carried out this program, Beatrice Gelber has since been largely forgotten. The sole photograph we have been able to trace of her comes from a news story (Figure 1). Our goal is not only to rehabilitate her scientific legacy, but also to amplify some of her visionary ideas about the molecular biology of memory.

## The contributions of Beatrice Gelber

In the late 1940s, Beatrice Gelber was a divorced mother of 3 living with her grandmother in Long Beach, NY (Census data for 1930–1940). After her youngest child departed for college, she decided to follow her curiosity and enrolled in the graduate Psychology program at Indiana University. Her advisor was Roland Clark Davis, a leading proponent of ‘psychophysiology’, which sought to establish links between physiological and psychological phenomena. Davis primarily conducted electrophysiological experiments on humans (he was a pioneer in the study of the galvanic skin response), but just down the hall B.F. Skinner was conducting experiments on learning in pigeons. This made for a rowdy work environment:

"[T]he lab had a distinctive odor, which came from the multichannel recorder, an early inkless polygraph. Electrically activated metal pens—old phonograph needles through which a sizable current was passed during recording—traced the signal onto thin tar paper that was coated with gunpowder. As the paper flew through the recorder at 50 mm/s , the powder exploded off, leaving a fine black tar recording—and the room filled with smoke… [T]he acrid smell of the recording paper mixed with the stench of clucking pigeons." (Gabbay and Stern, 2012, p. 445).

Amidst this cacophony, while simultaneously pursuing her thesis research on muscular contraction, Gelber somehow found time to train *Paramecia*. In this effort, she benefitted from interactions with Tracy Sonneborn’s laboratory in the Department of Biology. Sonneborn was trained by Jennings in the study of ciliates, specializing in the study of *Paramecia* and becoming one of the leading geneticists of the era before molecular biology (Sapp, 1987). He made pioneering contributions to the study of non-Mendelian forms of inheritance (Preer, 1996). In addition to Skinner and Sonneborn, Indiana University was inhabited at that time by a number of other important figures in biology and psychology: Salvador Luria (whose collaboration with Max Delbrück, showing that bacteria and phage had genes, earned them Nobel Prizes), Luria’s first graduate student James Watson (co-discoverer of the structure of DNA), and William Estes (a former Skinner student who became faculty, making important contributions to learning theory). It is conspicuous that Gelber’s interests occupied the epicenter of this intellectual cauldron. Yet she appeared to be *sui generis*. Davis’ son, Chris Davis, who followed his father into psychophysiology, worked with Gelber as an undergraduate student and recalls her being a demanding experimentalist as well as a pleasant, if formal, supervisor, who occupied a place within the department that was apart from both her fellow students and the faculty (personal communication). Gelber seems to have had considerable independence in her research while still a PhD student, reflecting perhaps both her own maturity and her supervisor’s encouraging mentorship (Gabbay and Stern, 2012).

**Figure 2.**
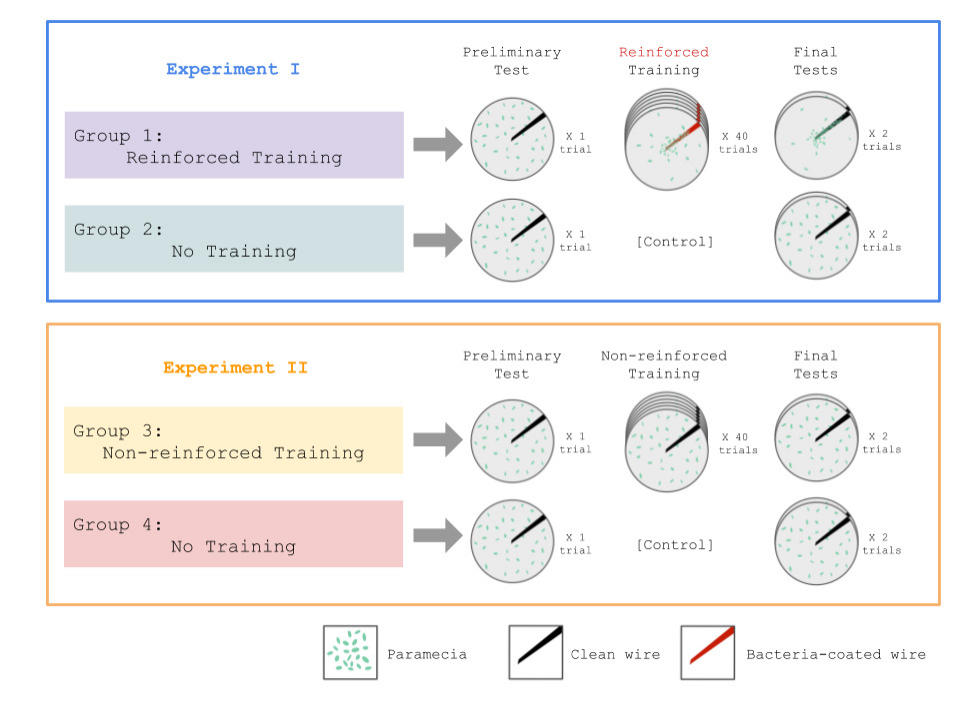
Design of experiments reported in Gelber, 1952. In Experiment 1 (top), one group of *Paramecia* was exposed to intermittent training trials in which a wire was coated with bacteria (every 3rd trial during the training phase). This group acquired a conditioned response to the clean wire, as measured by adherence to the wire in final test trials. In contrast, an untrained group did not show a conditioned response. Experiment 2 (bottom) demonstrated that the wire by itself did not drive conditioned responding.

Taking advantage of the fact that *Paramecia* feed upon bacteria, Gelber asked the following question: would *Paramecia* learn to approach a wire that was repeatedly coated with bacteria? In her first experiment, Gelber, 1952 compared one group of *Paramecia* who received reinforced training with another group that received no training (Figure 2, top). Each reinforced trial consisted of swabbing the wire with a bacterial suspension and then dipping it into the *Paramecia* culture for 15 s. Both groups were given preliminary and final tests, which consisted of dipping the wire without any bacteria. Gelber reported that the reinforced group clung to the wire in greater numbers on the test trials compared to the preliminary tests, and also compared to the no training group.

One problem with Gelber’s first experiment was that it did not control for repeated exposure to the wire itself as a driver of behavioral change. Accordingly, in her second experiment (Figure 2, bottom) she tested a non-reinforced training group that received wire-alone trials; this group did not show any evidence of conditioned responding. Thus, the combination of wire and bacteria was necessary to produce the learning effect.

It is worth noting here that Gelber was a very careful experimentalist. She knew that reorganization of nuclear material occurs during conjugation (sexual reproduction) and autogamy (self-fertilization) and was keen to avoid this because of her initial hypothesis that the memory trace (engram) was stored in the macronucleus (see Gelber and Rasch, 1956; Gelber, 1958). To accomplish her goal, she used a single mating type (eliminating conjugation) and small amounts of bacteria (suppressing autogamy). She also tracked the number of fissions, knowing that autogamy would occur after a certain number of fissions.

Having established the basic learning phenomenon, Gelber moved to the University of Chicago. She appears to have been a postdoctoral researcher in the Department of Psychology, judging by the affiliation given in her papers (Gelber, 1957), and again to have had substantial independence in formulating her own research. The Chicago Maroon describes her on 3 June 1955 as a NIH Postdoctoral Fellow in the Department of Physiology (which we presume to be a misprint for the Department of Psychology) and a recipient of NIH grant funding, in the same company as the psychologist Bruno Bettelheim and the biophysicist Aaron Novick.

Gelber proceeded to examine how a number of different factors influenced both learning and retention. In one of her only collaborative papers, she worked with Ellen Rasch to determine *Paramecium* DNA content and carefully analyzed the interplay between feeding, fission and autogamy (Gelber and Rasch, 1956). In her solo work, she first demonstrated that *Paramecia* would not approach the wire when tested in the dark (Gelber, 1956), indicating that they relied on photosensing to detect the conditioned stimulus in the absence of chemical gradients from the bacteria. Second, she demonstrated that *Paramecia* retained their memory 3 hr after spaced (long inter-trial interval) training but not after massed training (Gelber, 1958). This finding agrees with spacing effects observed in many animal species, ranging from bees (Menzel et al., 2001) to humans (Ebbinghaus, 1885; Cepeda et al., 2008). She acknowledged that this was a rather short retention interval compared to animal studies, but also noted that *Paramecia* can go through two reproductive cycles during this time, so it could be argued that 12 hr is a long period of time for a *Paramecium*. In later work, she showed that retention could last up to 12 hr (Gelber, 1962b), and other work using a different paradigm has shown retention up to 24 hr (Hennessey et al., 1979). Third, she observed a phenomenon known as *reminiscence* (Payne, 1987), where performance increases with moderate retention intervals before declining again, a more complex picture than the one classically given by monotonic forgetting functions. A recurring theme in these investigations was the parallel between learning in animals and *Paramecia*.

Gelber was repeatedly confronted by critics who denied that *Paramecia* were capable of sophisticated behavior. Donald Jensen, another psychologist working with *Paramecia*, argued that the increased rate of attachment to the wire as a consequence of training could be a form of ‘thigmotropism’ (attraction to a haptic stimulus) induced by the bacteria, possibly due to release of metabolic products like carbon dioxide or carbonic acid. He showed that addition of bacteria to a culture increased attachment to the bottom of the slide, but he did not demonstrate that bacteria increase attachment to the wire (which was not used at all in this experiment). Indeed, by the same logic we might expect thigmotropism to inhibit the learning effect, since the wire contacted the bottom of the depression slide, and therefore the *Paramecia* would be attracted to both the slide surface and the wire. In any case, Gelber, 1957 failed to replicate Jensen’s findings.

Jensen further argued that Gelber’s findings might be an artifact of changes to the culture induced by training, rather than any persistent change in the organism itself (Jensen, 1957a). In support of this claim, Jensen showed that samples taken from the vicinity of the wire contained large numbers of bacteria after training, in contrast to samples taken far away from the wire. In response, Gelber, 1957 pointed out that she obtained a learning effect despite routinely stirring the slide between trials, presumably diluting any residual concentration of bacteria around the wire. Jensen, 1957b retorted that this stirring method was not sufficient to disperse the bacteria. She also showed that inserting the wire with food after training produced a greater response than food alone, indicating that the wire was contributing something beyond the effect of the bacteria. Finally, she pointed out that Jensen deviated from her experimental procedures in a number of other ways, including using bacterial concentrations several magnitudes higher than she had used (a point brushed aside by Jensen without any justification), and introducing the bacteria into distilled water instead of culture fluid (“the amount of steak found in a dish placed on the floor in an empty room would be very different from the contents of the same dish with a hungry dog present.”). The difference in bacterial concentration may have resulted in a greater amount of residual bacteria after removal of the wire, thereby degrading the contingency between the wire and bacteria (see further discussion below). It is now understood, as it was not at the time of this controversy, that when the background frequency or concentration of US is as frequent as that in the presence of the CS, no conditioning occurs (Rescorla, 1988).

Gelber’s dog analogy struck a chord with Jensen:

"The use of this analogy symbolizes what is perhaps the most basic difference of opinion between Gelber and me. Gelber freely applies to Protozoa concepts (reinforcement and approach response) and situations (food presentation) developed with higher metazoan animals. I feel that such application overestimates the sensory and motor capabilities of this organism… If analogies are necessary, a more apt one might be that of an earthworm which crawls and eats its way through the earth, blundering onto food-rich soil and avoiding light, heat, and dryness. Gelber’s assertion loses its force when the blind, filter-feeding mode of life of Paramecia is considered." (Jensen, 1957b, p. 1341).

In a commentary on this exchange, Kellogg, 1958 remarked that Jensen’s worm analogy was poorly chosen, since there was ample evidence that worms can learn. Kellogg saw in the Gelber-Jensen debate a reflection of the problem that had vexed some of the greatest thinkers: where do we draw the line between ‘higher’ and ‘lower’ organisms? If learning is one of those ‘higher’ faculties, then the demonstration of learning in single-celled organisms contradicts Loeb’s (and Jensen’s) idea that the behavior of apparently ‘lower’ organisms like *Paramecia* can be explained by various kinds of tropisms, without reference to ‘higher’ faculties.

Shortly after the Gelber-Jensen debate, Katz and Deterline, 1958 replicated Gelber but again raised the issue of changes to the culture due to the presence of the bacteria. They arrived at this conclusion on the basis of an experiment in which the culture was vigorously stirred immediately after the final training trial, before an additional ‘post-stir’ test, finding that stirring eliminated all conditioned responding. Their interpretation was that stirring dispersed any residual concentration of bacteria in the vicinity of the wire, which they assumed was driving the response to the wire. However, there is another interpretation of this result: it is a form of *contingency degradation* (Rescorla, 1968). The stirring procedure effectively decorrelated the wire (CS) and bacteria (US). It is well-known from Rescorla’s experiments and many others that this procedure suppresses conditioned responding to the CS, even when holding fixed the number of CS-US pairings. Indeed, a later experiment by Hennessey et al., 1979 used Rescorla’s ‘truly random control’, which explicitly decorrelates the CS and the US, finding that this eliminates learning of an avoidance response to a vibratory CS. It is also important to point out that procedurally, Katz and Deterline deviated in an important way from Gelber by measuring the number of bacteria in the vicinity of the wire rather than the number adhered to the wire, which means that in effect they were partially measuring ‘contextual’ conditioning.

Gelber’s subsequent career is hard to track after the lapse of years. The news story in Figure 1, from the Tucson Daily Citizen of 19 October 1960, describes her as a ‘refugee’ from the University of Chicago, holding a contract of $100,000 from the NIH and launching an institute for ‘off beat’ science. As to what became of this venture, we have no clue. In 1964, Gelber presented her *Paramecium* work at the symposium on ‘Learning and Associated Phenomena in Invertebrates’ in Cambridge, England (Thorpe and Davenport, 1965; McConnell, 1966). In 1969, she conducted a pilot study for the National Institute of Mental Health on what retired scientists do, having become by then one of her own subjects. She died in 1991. The Wikipedia entry on her arose from our enquiries. We would be delighted to know from any readers who can add to the limited story of her life that we have been able to piece together.

Taking stock, we believe that Gelber’s experiments, though not without their limitations, convincingly demonstrated Pavlovian conditioning in *Paramecia*. Sadly, her critics seem to have won in the long term. Most reviews of the literature, if they mention Gelber’s work at all, quickly dismiss it on the basis that it was confounded by more plausible alternative explanations (e.g. Warren, 1965; Applewhite, 1979). An exception is the review of invertebrate learning by McConnell, 1966, who was sympathetic to Gelber’s conclusions. In discussing the Katz and Deterline stirring result, he wryly noted that “since these authors failed to control for the effects of ‘vigorous stirring’ on the behavior of *Paramecia*, and since even an overtrained rat that was ‘well shaken’ right after a final training trial might choose not to run the maze if returned to it immediately, the issue cannot be resolved on the basis of these studies alone’ (p. 112). McConnell, 1966 himself showed conditioning in planaria; his reports of RNA-based memory transfer during regeneration and cannibalism provoked a controversy to which we return in the next section.

In surveying much of this work, it is hard not to feel that participants divided into those opposed to the possibility of learning in single-cell organisms and those sympathetic to the possibility and that their interpretations were strongly influenced by these prejudgements. One of the challenges in revisiting these controversies is how to formulate experiments which can transcend ideological stances and elicit compelling scientific insights.

## Implications for the neurobiology of learning and memory

If single cells can learn then they must be using a non-synaptic form of memory storage. The idea that intracellular molecules store memories has a long history, mainly in the study of multicellular organisms. We have already mentioned McConnell’s studies of planarians; similar ideas were espoused by Georges Ungar based on his studies of rodents (Ungar and Irwin, 1967; Ungar et al., 1968). These studies indicated that memories could be transferred from one organism to another by injection or ingestion of processed brain material. Clearly no synaptic information could survive such processing, so transfer could presumably only occur if the memory substrate was molecular. However, these findings were the subject of much controversy. The failure of careful attempts to replicate them led to a strong consensus against their validity and this line of research eventually died out (Byrne et al., 1966; Travis, 1980; Smith, 1974; Setlow, 1997). Nonetheless, several lines of recent work have revisited these studies (Smalheiser et al., 2001; Shomrat and Levin, 2013). For example, Bédécarrats et al., 2018 showed that long-term sensitization of the siphon-withdrawal reflex in *Aplysia* could be transferred by injection of RNA from a trained animal into an untrained animal. This study further showed that this form of transfer was mediated by increased excitability of sensory (but not motor) neurons, and depended on DNA methylation, although the study did not establish either RNA or DNA methylation as the engram storage mechanism. In another line of work, Dias and Ressler, 2014 showed that fear conditioning in rodents could be transferred from parents to offspring, an effect that was associated with changes in DNA methylation. These studies not only revive the molecular memory hypothesis, but also point towards specific intracellular mechanisms.

The significance of DNA methylation lies in the fact that DNA methylation state can control transcription. Thus, the set of proteins expressed in a cell can be altered by changes in DNA methylation, which are known to occur in an experience-dependent manner. For example, after fear conditioning, the methylation states of 9.2% of genes in the hippocampus of rats were found to be altered (Duke et al., 2017). As first pointed out by Crick, 1984, and later elaborated by Holliday, 1999, DNA methylation is a potentially stable medium for heritable memory storage, because the methylation state will persist in the face of DNA replication, thanks to the semi-conservative action of DNA methyltransferases. A related idea, put forward independently in Lisman et al., 2018, is that a stable memory could arise from the tug-of-war between enzymatic phosphorylation and dephosphorylation. In essence, the idea is to achieve stability through change: a molecular substrate maintains its activation state by means of continual enzymatic activity. Crick and Lisman suggested that this could solve the problem of molecular turnover that vexes synaptic theories of memory. Consistent with this hypothesis, inhibition of DNA methyltransferase disrupts the formation and maintenance of memory, although it remains to be seen whether methylation states themselves constitute the engram (Miller and Sweatt, 2007; Miller et al., 2010). The proposals of Crick and Lisman apply generally to enzymatic modification processes (e.g. acetylation or glycosylation) acting on macromolecules, provided that the biochemical dynamics can generate the appropriate stable states (Prabakaran et al., 2012).

An important distinction between the forms of dynamical information storage proposed by Crick and Lisman and the storage provided by DNA is that the latter is largely stable in the absence of enzymatic activity, under conditions of thermodynamic equilibrium. In contrast, the former typically relies on enzymatic activity and is only stable if driven away from thermodynamic equilibrium by chemical potential differences generated by core metabolic processes. In other words, the latter may accurately retain information in the absence of a cell over a substantially longer period than the former, which may lose information rapidly in the absence of supporting enzymatic activity.

Another candidate medium for intracellular memory storage is histone modification. In eukaryotes, DNA is wrapped around nucleosomes, composed of histone proteins, to form chromatin. Gene transcription can be controlled by changes in the modification state (acetylation, methylation, ubiquitination, etc.) of these histones. In the cell biology literature, an influential hypothesis posits the existence of a histone ‘code’ (Jenuwein and Allis, 2001; Turner, 2002) or ‘language’ (Lee et al., 2010) that stores information non-genetically, although the nature of that information has been a matter of debate (Sims and Reinberg, 2008; Henikoff and Shilatifard, 2011). Early work demonstrated that learning was accompanied by increased histone acetylation in the rat hippocampus (Schmitt and Matthies, 1979), and more recent work has established that memory can be enhanced by increases in histone acetylation (Levenson et al., 2004; Vecsey et al., 2007; Stefanko et al., 2009). Bronfman et al., 2016 provide an extensive survey of the molecular correlates of learning and memory.

In parallel with these findings, molecular biologists grappling with the information processing that takes place within the organism have begun to suggest that signaling networks may implement forms of learning (Koseska and Bastiaens, 2017; Csermely et al., 2020). In this respect, Koshland’s studies of habituation of signaling responses in PC12 cells, a mammalian cell line of neuroendocrine origin, are especially resonant (McFadden and Koshland, 1990). Koshland’s work was undertaken in full awareness of learning studies conducted by Kandel and Thompson in animals but his pioneering efforts have not been explored further. This reflects, perhaps, the intellectual distance between cognitive science and molecular biology, which the present paper seeks to bridge. The information processing demands on a single-celled organism, which must fend for itself, are presumably quite different from those confronting a single cell within a multi-cellular organism during development and homeostasis, so what role learning plays within the organism remains a tantalizing open question.

Beatrice Gelber, though she could not have known about the specifics of DNA methylation or histone modification, was uncannily prophetic about these developments:

"This paper presents a new approach to behavioral problems which might be called molecular biopsychology… Simply stated, it is hypothesized that the memory engram must be coded in macromolecules… As the geneticist studies the inherited characteristics of an organism the psychologist studies the modification of this inherited matrix by interaction with the environment. Possibly the biochemical and cellular physiological processes which encode new responses are continuous throughout the phyla (as genetic codes are) and therefore would be reasonably similar for a protozoan and a mammal." (Gelber, 1962a, p. 166).

The idea that intracellular mechanisms of memory storage might be conserved across phyla is tantalizing yet untested. The demise of behavioral studies in *Paramecia* and other ciliates has meant that, despite the wealth of knowledge about ciliate biology, we still know quite little about the molecular mechanisms underlying Gelber’s findings. Nonetheless, we do know that many intracellular pathways that have been implicated in multicellular memory formation exist in ciliates (Table 1). For example, ciliates express calmodulin, MAP kinases, voltage-gated calcium channels, in addition to utilizing various epigenetic mechanisms that might be plausible memory substrates, such as DNA methylation and histone modification. In like manner, key molecular components of neurons and synapses emerged in organisms without nervous systems, including unicellular organisms (Ryan and Grant, 2009; Arendt, 2020). We believe it is an ideal time to revisit the phylogenetic origins of learning experimentally and theoretically.

**Table 1.** Molecules/pathways known to be involved in learning and memory, with homologues in ciliates.

Molecules/pathways known to be involved in learning/memory	Ciliates with reported homologues	References
N-methyl-D-aspartate receptor (NMDAR)	*P. primaurelia* (only partial sequences)	Ramoino et al., 2014
Glutamate receptor	*P. tetraurelia*	Van Houten et al., 2000
Calmodulin	*P. tetraurelia*	Plattner and Verkhratsky, 2018
cAMP	*P. tetraurelia*	Plattner and Verkhratsky, 2018
cAMP-dependent protein kinase	*P. tetraurelia*	Plattner and Verkhratsky, 2018
Mitogen activated protein kinase (MAPK)	*P. caudatum*	Wada and Watanabe, 2007
Protein kinase C (PKC)	*T. thermophilia*	Hegyesi and Csaba, 1994
Calcineurin	*P. tetraurelia*	Plattner and Verkhratsky, 2018
DNA methyltransferases (DNMTs)	*T. thermophilia*	Gutiérrez et al., 2000
Histone acetyltransferases (HATs)	*T. thermophilia*	Vavra et al., 1982
Histone deacetylases (HADCs)	*T. thermophilia*	Wiley et al., 2005

## Conclusion

Single cells continue to surprise us. Robert Hooke, peering through his microscope in the 17th century, first likened cells to the small rooms (cellula) inhabited by monks. Fast forward to the 21st century, and it is now banal for cell biologists to think of the cell as a miniature computer, capable of sophisticated information processing (Bray, 2009). Among their many capabilities, it is now appreciated that cells have memory, possibly in the form of a ‘histone code’ (Jenuwein and Allis, 2001; Turner, 2002), though a precise computational understanding of this code has remained elusive. Whatever the memory code may be, its implications for neuroscience are far-reaching: we may finally be poised to link cellular memory codes with cognitive information processing. In this context, the studies by Gelber and others of learning in *Paramecia* become freighted with significance. They suggest that single cells have the ability to carry out a form of information processing that neuroscientists have traditionally attributed to networks of cells. We still do not understand how *Paramecia* accomplish this feat. If the hypothesis is correct, then single cells hold more surprises in store for us.
